# Hydrogen Sulfide Modulates Astrocytic Toxicity in Mouse Spinal Cord Cultures: Implications for Amyotrophic Lateral Sclerosis

**DOI:** 10.3390/antiox13101241

**Published:** 2024-10-15

**Authors:** Susanna De Stefano, Marta Tiberi, Illari Salvatori, Marco De Bardi, Juliette Gimenez, Mahsa Pirshayan, Viviana Greco, Giovanna Borsellino, Alberto Ferri, Cristiana Valle, Nicola B. Mercuri, Valerio Chiurchiù, Alida Spalloni, Patrizia Longone

**Affiliations:** 1 Department of Systems Medicine, University of Rome Tor Vergata, 00133 Rome, Italy; destefanosusanna@gmail.com (S.D.S.); m.tiberi@hsantalucia.it (M.T.); mercurinb@gmail.com (N.B.M.); 2 Laboratory of Molecular Neurobiology, IRCCS Santa Lucia Foundation, 00143 Rome, Italy; j.gimenez@hsantalucia.it (J.G.); mahsa.pirshayan@students.uniroma2.eu (M.P.); a.spalloni@hsantalucia.it (A.S.); 3 Laboratory of Resolution of Neuroinflammation, IRCCS Santa Lucia Foundation, 00143 Rome, Italy; v.chiurchiu@hsantalucia.it; 4 Laboratory of Neurochemistry, IRCCS Santa Lucia Foundation, 00143 Rome, Italy; i.salvatori@hsantalucia.it (I.S.); alberto.ferri@cnr.it (A.F.); cristiana.valle@cnr.it (C.V.); 5 Neuroimmunology Unit, IRCCS Santa Lucia Foundation, 00143 Rome, Italy; m.debardi@hsantalucia.it (M.D.B.); g.borsellino@hsantalucia.it (G.B.); 6 Fondazione Policlinico Universitario Agostino Gemelli IRCCS, 00168 Rome, Italy; vivianagreco82@yahoo.it; 7 Institute of Biochemistry and Clinical Biochemistry, Università Cattolica del Sacro Cuore, 00168 Rome, Italy; 8 Institute of Translational Pharmacology (IFT), National Research Council (CNR), 00133 Rome, Italy; 9 Laboratory of Experimental Neurology, Santa Lucia Foundation IRCCS, 00143 Rome, Italy

**Keywords:** hydrogen sulfide, astrocytes, mitochondria, motor neuron

## Abstract

Hydrogen sulfide (H_2_S), a known inhibitor of the electron transport chain, is endogenously produced in the periphery as well as in the central nervous system, where is mainly generated by glial cells. It affects, as a cellular signaling molecule, many different biochemical processes. In the central nervous system, depending on its concentration, it can be protective or damaging to neurons. In the study, we have demonstrated, in a primary mouse spinal cord cultures, that it is particularly harmful to motor neurons, is produced by glial cells, and is stimulated by inflammation. However, its role on glial cells, especially astrocytes, is still under-investigated. The present study was designed to evaluate the impact of H_2_S on astrocytes and their phenotypic heterogeneity, together with the functionality and homeostasis of mitochondria in primary spinal cord cultures. We found that H_2_S modulates astrocytes’ morphological changes and their phenotypic transformation, exerts toxic properties by decreasing ATP production and the mitochondrial respiration rate, disturbs mitochondrial depolarization, and alters the energetic metabolism. These results further support the hypothesis that H_2_S is a toxic mediator, mainly released by astrocytes, possibly acting as an autocrine factor toward astrocytes, and probably involved in the non-cell autonomous mechanisms leading to motor neuron death.

## 1. Introduction

Hydrogen sulfide (H_2_S) is a small molecule that can freely travel through cell membranes. Similarly to other gaseous molecules with known biological functions, like nitric oxide (NO) and carbon monoxide (CO), it is a physiologically important transmitter that is particularly abundant in the brain [1]. H_2_S is produced in mammalian tissues through an endogenous synthetic system that consists primarily of three enzymes, cystathionine β-synthase (CBS), cystathionine γ-lyase (CSE), and mercapto-pyruvate sulfur transferase (MPST; also referred to as MST), through the cysteine transamination product 3-mercaptopyruvate [1,2,3]. It is endogenously produced in the brain, mainly in astrocytes [4,5], although the neuronal contribution to its production appears to be fundamental, since CBS expression and H_2_S production are influenced by the presence of neurons [6].

In physiological conditions, endogenous H_2_S is not toxic to cells [7,8]. However, depending on its concentration, it may have a beneficial or adverse role [9]. In mitochondria, like cyanide, it directly inhibits cytochrome c oxidase (Complex IV), blocking respiration and ATP synthesis [10] and inducing substantially more oxidative stress than cyanide [11]. It inhibits oxidative-stress-induced apoptosis at low doses, while a relatively higher dose aggravates the ailment by promoting the same pathway [12]. In cultured astrocytes from the spinal cord, it induces Ca^2+^ release from mitochondria and the endoplasmic reticulum, provoking an increase in lactate production [5,13].

In our previous work, we measured poisonous concentrations of H_2_S in the liquor of patients affected by Amyotrophic Lateral Sclerosis (ALS), a neurodegenerative disease with sporadic or familial forms, characterized by the progressive death of spinal and cortical motor neurons and the involvement of non-neuronal cells, such as astrocytes [14,15]. In the same work, we also demonstrated that H_2_S is toxic to motor neurons and that its medium concentration decreases when astrocyte proliferation is halted by arabinoside-C (Ara-C) [16]. Furthermore, we also showed [14] that its concentration is increased in primary spinal cord cultures prepared from a genetic ALS mouse model, using SOD1G93A mice, carrying a point mutation at position 93 in the superoxide dismutase 1 protein, which is linked to a familial form of ALS [17].

Here, we sought to further investigate the effects of H_2_S on primary spinal cord cultures by evaluating whether H_2_S modulates glial cell phenotypes and morphology as well as its metabolic function on mitochondrial bioenergetics.

## 2. Material and Methods

### 2.1. Spinal Cord Culture and Treatments

Mixed spinal cord cultures were prepared largely as previously described [18] from 13.5-day-old C57BL/6J embryos. Animal care and use followed the European Directive (2010/63/EU) adopted by the Council of the European Union for animal experiments and adequate measures were taken to minimize pain or discomfort. The experimental protocol was approved by the Italian Ministry of Health (license Number n° 536/2022-PR). Each neural tube was dissected singularly, and the resulting mixed cultures were seeded on poly-L-lysine–coated glass cover slips (3 cover slips for each spinal cord/dish). All the experiments were performed on spinal cord cultures between 10 and 12 days in vitro (DIV), unless otherwise indicated. In dedicated experiments, glial proliferation was halted 3 days after seeding with the addition of arabinoside C (Ara-C; 10 µM). Spinal cord cultures were exposed to 200 μM of the H_2_S donor Na_2_S. The concentration used was the one showing the highest toxicity, as we have previously demonstrated [14,19].

### 2.2. Immunophenotyping by Flow Cytometry

Cellular phenotypes were assessed using multiparametric flow cytometry panels containing markers to identify cell types and markers to assess activation states. The use of these markers allowed us to identify all cells of interest based on physical parameters (side and forward scatter) and by using specific antibodies. For the immunophenotyping of the spinal cord mixed primary culture, cells were stained with different panels of cell surface markers (see Table 1) [20,21].

Astrocytes were identified as ACSA-2^+^ cells, microglia as CD45^low^CD11b^+^ cells, oligodendrocytes as O4^+^ cells, endothelial cells as CD31^+^ cells, and neurons as CD90.2^+^ cells. Also, infiltrated leukocytes were identified as CD45^high^CD11b^+^ cells.

Another panel was used for the identification of reactive A1 astrocytes as ACSA-2^+^C3^+^ cells, whereas non-reactive A2 astrocytes were identified as ACSA-2^+^S100A^+^ cells. All samples were acquired on a 13-color Cytoflex (Beckman Coulter, Brea, CA, USA) device, and for each analysis, at least 0.5 × 10^6^ living cells were acquired.

### 2.3. Immunofluorescence

Cultures were then fixed with PFA 4% and stained with the appropriate primary antibody (listed in Table 1). Confocal images were captured at 10Χ, 20Χ, and 63Χ using a Zeiss LSM800 confocal laser-scanning microscope (Carl Zeiss, Jena, Germany). Images were acquired in the sequential scanning mode by using the same acquisition parameters for all the images (laser intensities, gain photomultipliers, pinhole aperture). For visualization purposes, channel colors were palette-assigned and reflected the true fluorochrome color. For the production of figures, the brightness and contrast of the images were adjusted by taking care to leave a light fluorescence background for visual appreciation of the lowest-fluorescence-intensity features and to help in the comparison among the different experimental groups. The ImageJ software (V1.8.0) (NIH, Bethesda, MD, USA) [22] was used to determine pixels values. The soma diameter was calculated using the ImageJ software.

### 2.4. Morphological Assessment

Mixed spinal cord cultures were stained with GFAP, and the soma size of non-overlapping astrocytes was measured using the ImageJ analysis system [22]. On each image at a 10 X zoom with 1 stack size, X = 512 mm, and y = 512 mm, using the proprietary Zeiss LSM image analyzer program, a scale bar was traced along the diameter of the GFAP+ cells in the non-treated cultures. The same image was used to set the measurement scale on ImageJ using the microM of the Zeiss LSM scale bar as a reference. Measurements were made on the images at the same magnification. Then, different fields per condition were analyzed, and at least 10 cells per field were measured. A person blind to the treatment performed the analyses.

### 2.5. Protein Extraction and Western Blot

Cells were washed twice in PBS and homogenized in RIPA buffer (150 mM NaCl, 1.0% IGEPAL, 0.5% sodium deoxycholate, 0.1% SDS, 50 mM Tris, pH 8.0, with a complete cocktail of proteases and phosphatases), followed by centrifugation (12,000 rpm, 10’) to pellet the cell debris and separate the supernatant [23]. Protein concentration was estimated using a Bradford assay with bovine serum albumin as a standard. A total of 50 μg of soluble per lane protein was loaded on 12% SDS-polyacrylamide gel and transferred to a PVDF membrane. After blotting, non-specific binding was blocked by 5% non-fat dry milk in PBS 1% Tween and incubated overnight at 4 °C with the following primary antibodies: anti-caspase-3 (1: 100; Invitrogen), anti-connexin 43 (1:500; Sigma), and anti-cytocrome C (1:100; Invitrogen) diluted into 5% non-fat dry milk in PBS and 1% Tween. As a loading control anti-GAPDH (1: 60,000; Immunological Sciences) was used (Table 1).

The membrane was washed and developed with Western blotting chemiluminescence luminol reagent (Thermo Fisher Scientific, Milan, Italy) following the manufacturer’s instructions. The optical density (O.D.) of each band was divided by the corresponding O.D. for GAPDH. The resulting ratio was compared to the one obtained from culture extracts that were not treated with H_2_S and plotted as percentage.

### 2.6. JC-1 and TMRM Dye Staining

To determine the mitochondrial membrane potential (Δ*Ψ*m) in cultured neurons, we employed the mitochondrial membrane indicator dye JC-1 (tetraethylbenzimidazolylcarbocyanine iodide) and the potentiometric, cell-permeable fluorescent probe TMRM (tetramethylrhodamine, methyl ester) [24,25]. The TMRM signal is directly correlated with the Δ*Ψ*m value across the inner mitochondrial membrane; healthy cells with functioning mitochondria have a bright TMRM signal, and when mitochondria lose their membrane potential, the TMRM signal dims or disappears. The JC-1 signal shifts from green to red with increasing aggregation in mitochondria, thus allowing for a ratiometric, quantitative assessment of the mitochondrial polarization states. After Na2S treatment, the cultures were loaded with either TMRM (Molecular Probes, 200 nM) or JC-1 (Molecular Probes, 2 µg/mL) for 30 min at 37 °C and then fixed and analyzed with the Zeiss LSM800 confocal laser-scanning microscope (Carl Zeiss, Germany) as described above.

### 2.7. Mitochondrial Bioenergetics

Evaluation of the oxygen consumption rate (OCR) of the cells was performed using a Seahorse XF96 Analyzer (Seahorse Bioscience–Agilent, Santa Clara, CA, USA). Mitochondrial functions were analyzed through a Seahorse XF Cell Mito Stress Test kit (Seahorse Bioscience) following the manufacturer’s protocol and according to Salvatori et al. [26]. Briefly, spinal cord neurons were placed into individual wells in the appropriate Seahorse Cell Culture Microplates (Agilet Seahorse XF96). A Cell Mito Stress Test was performed on the cell cultures incubated for 45 min in a CO_2_-free incubator at 37 °C for 45 min after replacement the medium with XF Base (Agilent) supplemented with 1 mM pyruvate, 2 mM glutamine, and 10 mM glucose. Basal respiration was calculated after subtraction of the non-mitochondrial respiration; ATP production was calculated following the addition of oligomycin. Maximal respiration was measured following the addition of FCCP (carbonyl cyanide-p-trifluoromethoxyphenylhydrazone); spare respiration capacity was calculated as the difference between maximal and basal OCR.

### 2.8. Cell Sorting and Isolation of ACSA-1 and 2+ Cells

Mouse primary spinal cord cultures were resuspended in 1 × PBS-0.5% BSA, stained with 1 μL of propidium iodide (PI, SIGMA P4864) for dead cell exclusion, and filtered with a 50 μm pore filter (BD Biosciences) to remove debris and cell clumps. To identify the astrocytes, we probed the cultures with the ACSA-1 and -2 surface markers. Cells were sorted with a MoFlo Astrios EQ (Beckman Coulter) device directly into ice-cold lysis buffer (Reliaprep RNA Cell Miniprep System, Promega, Fitchburg, WI, USA), mixed by vortexing, and then stored at −80 °C until RNA extraction.

### 2.9. RNA Extraction and qRT-PCR

RNA was isolated using TRIzol Reagent (Ambion by life technologies, Carlsbad, CA, USA). The pellet was resuspended in 15 μL of RNAase-free water and quantified using a Nanodrop 2000c spectrophotometer (Thermo scientific) [19,23].

Following the reverse transcription of 80 ng RNA (Table 2), real-time PCR was carried out on a Light Cycler 480 (Roche Diagnostics, Monza, Italy) device using the SYBR green method.

A total of 2 μL of cDNA product was used as a template in a 20 μL final PCR reaction containing 10 μL of SYBR GREEN (LightCycler 480 sybr green I master, Roche), 2 μL of each primer (their concentrations were 5 μM), and 6 μL of RNAse-free distilled water. All reactions were carried out in duplicate. Amplification protocols were as follows: 95 °C for 3 min; 45 cycles of 95 °C/10 s, 60 °C/20 s, and 72 °C/10 s; and 1 cycle of 95 °C/10 s and 60 °C/10 s for melting curve analysis. The threshold cycle number (Ct) was automatically determined by the LightCycler 480 system (Roche). L34 was used as the housekeeping gene, and the primers were from Ramljak et al. [27].

### 2.10. Statistics

All quantitative data were collected using Microsoft Excel 365 (Office16) spreadsheets. Where appropriate, the data were normalized to the values of the untreated control cultures. Student’s *t*-test was used for real-time analysis. For the study of *mitochondrial bioenergetics*, protein expression, and Cx 43 real-time analyses, one-way ANOVA with Tukey’s test was used to test for statistical significance. Independent experiments were repeated at least three times (unless otherwise stated). All flow cytometry analyses were performed with the FlowJo 10.7.1 software program (Treestar, Ashland, OR, USA). Data were presented as mean ± SEM, and changes were considered significant with a *p*-value of ≤0.05. *p*-values ≤ 0.05 are marked with *, *p*-values ≤ 0.01 are marked with **, and *p*-values ≤ 0.001 are marked with ***.

## 3. Results

### 3.1. Hydrogen Sulfide Affects Astrocyte Morphology and Activation Status

**Figure 1:**
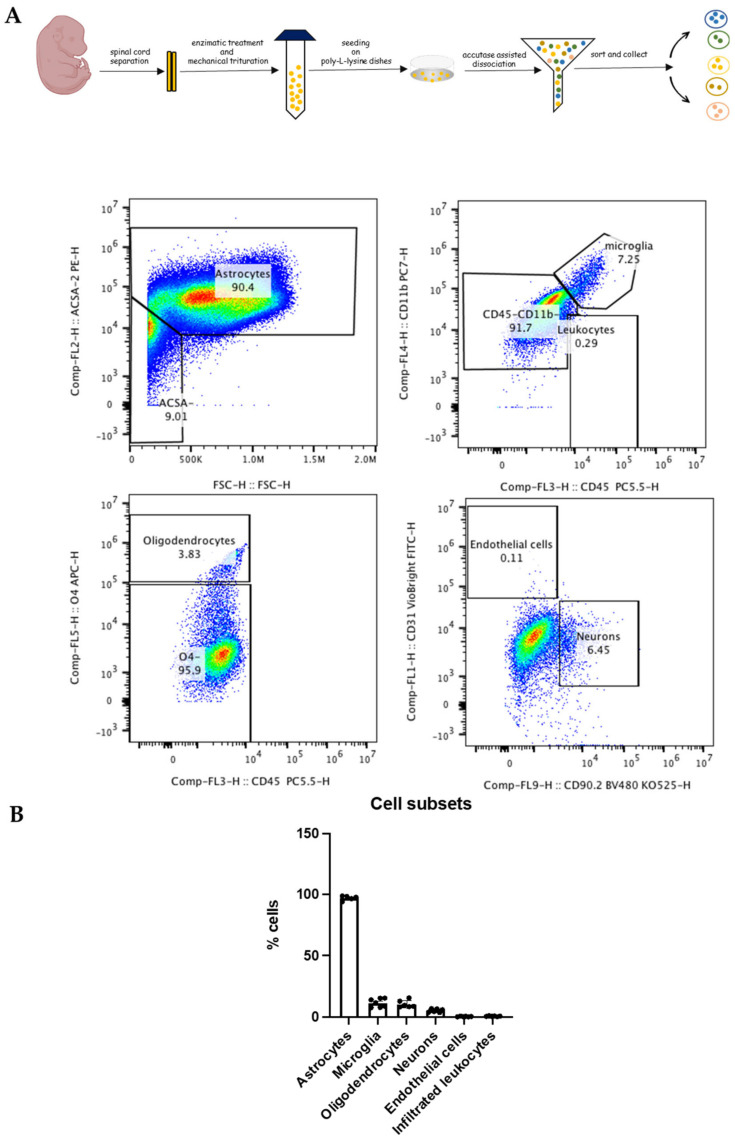
Astrocytes are the major non-neuronal cell population in primary spinal cord cultures. (**A**) Spinal cord cultures were prepared from 13.5 day-old embryos, and after 10–12 days in cultures, their composition was analyzed by high-dimensional flow cytometry using specific cellular markers (see the Section 2.2). (**B**) The flow cytometry analyses attested that astrocytes were the major non-neuronal cell population in the spinal cord cultures (ACSA-2^+^).

Since our studies and those from others [5,14] have shown that H_2_S is released by astrocytes, herein, we wondered whether it would affect astrocyte survival and homeostasis by inducing morphological alterations or promoting the transition of astrocytes to a different phenotype. Firstly, by means of high-dimensional flow cytometry, we immunophenotyped spinal cord primary cultures (Figure 1A) in order to deeply characterize all of the cell subsets, and we observed that ACSA-2^+^ astrocytes were the main glial population, representing over 90% of the mixed culture, followed by an equal 4% each of CD45^low^CD11b^low^ microglia and O4^+^ oligodendrocytes. The remaining 2% was represented by very few CD31^+^ endothelial cells and CD45^high^CD11b^high^ leukocytes (Figure 1B). For the neuronal count, we used surface CD90.2.

**Figure 2:**
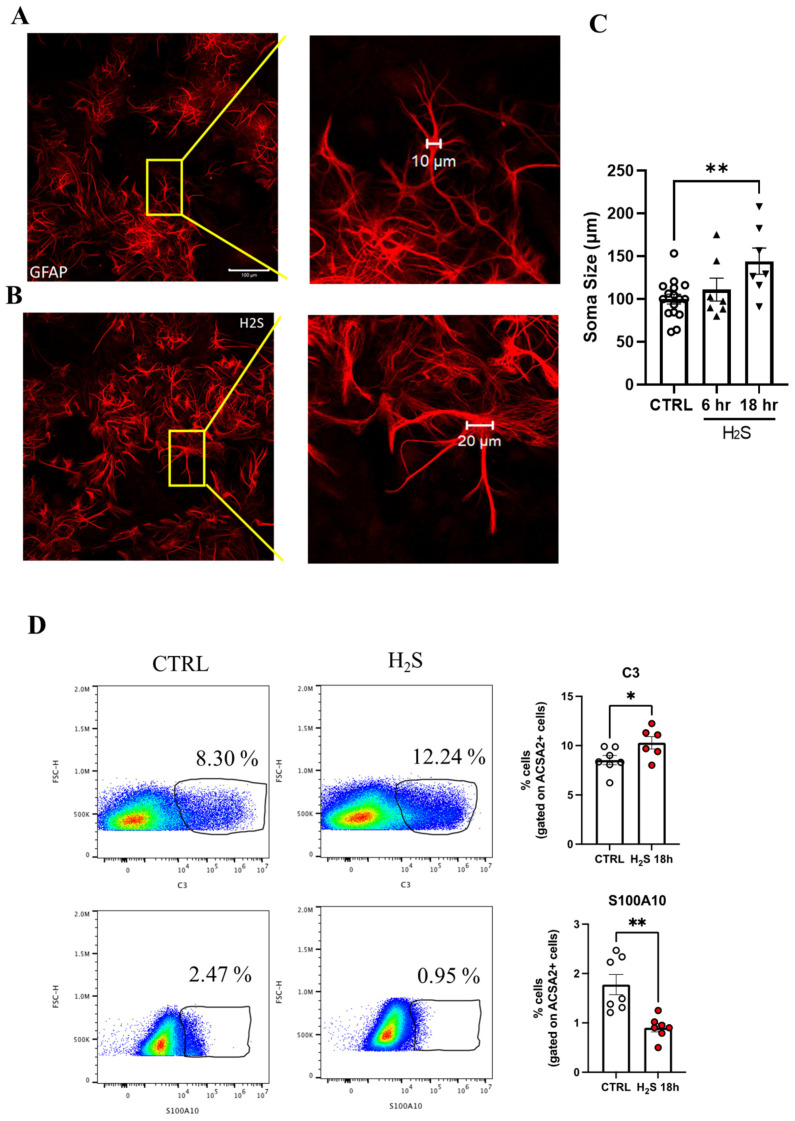
H_2_S affects astrocyte morphology and phenotype. Representative images of astrocytes (GFAP+) in (**A**) control (Ctrl) and (**B**) H_2_S-treated (200 µM, 18 h) cultures. (**C**) Cumulative data of the quantification of soma size at the indicated times. The size of the GFAP^+^ cell body was calculated by tracing a scale bar along the cell diameter in the control (10 µm) and treated (20 µm) cultures, where 10 random fields were captured, and 10 cells/fields were measured. (**D**) High-dimensional flow cytometry of A1 ACSA-2^+^ cell (C3^+^) and A2 ACSA-2^+^ (S100A^+^) markers. On the left are representative scatter plots of the flow cytometry analysis of spinal cord cultures treated with H_2_S; on the right are the cumulative data of the percentage of the C3^+^ and S110A^+^ cells in the control (Ctrl) and H_2_S-treated cultures. Data represent the mean ± SEM of three independent experiments, * *p* < 0.05 vs. CTRL, ** *p* < 0.01 vs. CTRL. In the (**C**) circles and triangles represent different time of treatments, control, 6, 18 h. In (**D**) white and red circles represent control and H_2_S (200 µM) 18 h.

Nevertheless, in order to investigate the effect of H_2_S on astrocytes, we performed a time course treatment (200 μM; at 3, 6, and 18 h), and our cytofluorimetric analysis revealed that the overall number of ACSA-2^+^ astrocytes was unaffected (Supplementary Figure S1A), while it showed a time-dependent increased toxicity towards CD90.2^+^ neurons, reaching a significant effect at 18 h. We further confirmed its toxicity to motor neurons by direct counting of the effect on SMI32+ neurons due to the sulfur component of the molecule and not to the saline one, as can be seen from the comparison with NaCl (Supplementary Figure S1B) and in agreement with our previous data [14,18]. We then assessed whether H_2_S had an effect on the astrocytic morphological features, which are known to be linked to astrocyte reactivity. Following the H_2_S challenge, the astrocytic cellular morphology was estimated by directly counting three characteristic astrocyte morphologies: arborized, polarized, and fibroblast-like [28]. Although the H_2_S-treated astrocytes did not show statistically different morphological alterations at any time points (Supplementary Figure S2A,B) nor did the expression of GFAP show any significant changes (Supplementary Figure S2C), the cells displayed an increased mean cell area from 6 μm (control) up to 10 to 12 μm, which was significant at 18 h (Figure 2A–C). We next sought to explore whether H_2_S had the potential to induce the transition between the A1/A2 astrocytic phenotypes by evaluating the percentages of the C3 “A1” type marker and the S100A10 “A2” type marker. High-dimensional flow cytometry revealed that H_2_S significantly increased the C3^+^ cells and significantly decreased the S100A10^+^ cells (Figure 2D). These analyses suggest that H_2_S, despite not affecting the total number of GFAP+ astrocytes, promotes their transformation towards the A1 reactive phenotype.

### 3.2. Cx43 Expression Is Increased in Spinal Cord Cultures Challenged with Hydrogen Sulfide

**Figure 3:**
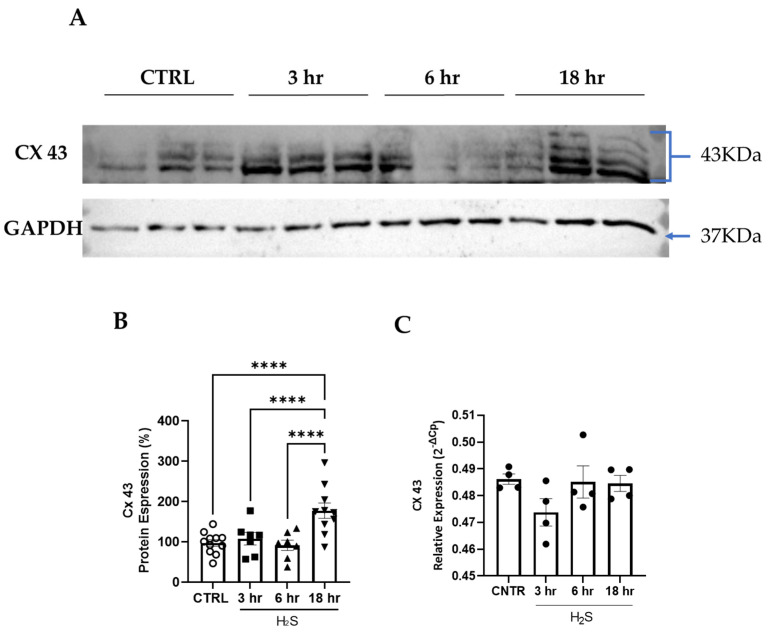
H2S treatment upregulates Cx43 expression. The expression levels of the Cx43 protein were analyzed by Western blotting following treatment with H_2_S (200 µM) at 3, 6, and 18 h. (**A**) Representative Western blot showing Cx43 protein expression in non-treated group (CTRL) and after H2S treatment. The expression of all the bands probed with the CX43 antibody were quantified and normalized to that of GAPDH, used as an internal loading control. (**B**) The data are expressed in percentages as the ratio of the sum of the Cx43 bands to GAPDH compared to the CTRL cultures and represent the mean ± SEM from seven to ten independent experiments, **** *p* < 0.0001 vs. CTRL. (**C**) The mRNA expression of Cx43 was assessed in the whole culture and remained unchanged throughout the time course. The values are represented as mean ± SEM of four independent experiments. For the protein expression circles, squares and triangles represent different time of treatments, control, 3, 6, 18 h.

Astrocytes form an interconnected network through a family of gap junction proteins known as connexins (Cxs). The main Cx in astrocytes is Cx43 [29]. Hence, we verified whether the H_2_S treatment affected Cx43 expression. A steady increase in Cx43 protein expression was found in the H_2_S-challenged cultures, reaching significant values at 18 h (Figure 3A,B), where the antibody against Cx43 detected at least 4 isoforms (Figure 3A), while the mRNA expression did not change (Figure 3C). Cx43 plays a fundamental role in astrocyte-to-astrocyte gap junctional communication [30].

### 3.3. Mitochondrial Function Is Impaired by H_2_S in Primary Spinal Cord Cultures

Given that the primary target of H_2_S toxicity is generally considered cytochrome c oxidase [11,31], the terminal enzyme of the mitochondrial oxidative phosphorylation, we sought to assess the mitochondrial function during H_2_S treatment.

**Figure 4:**
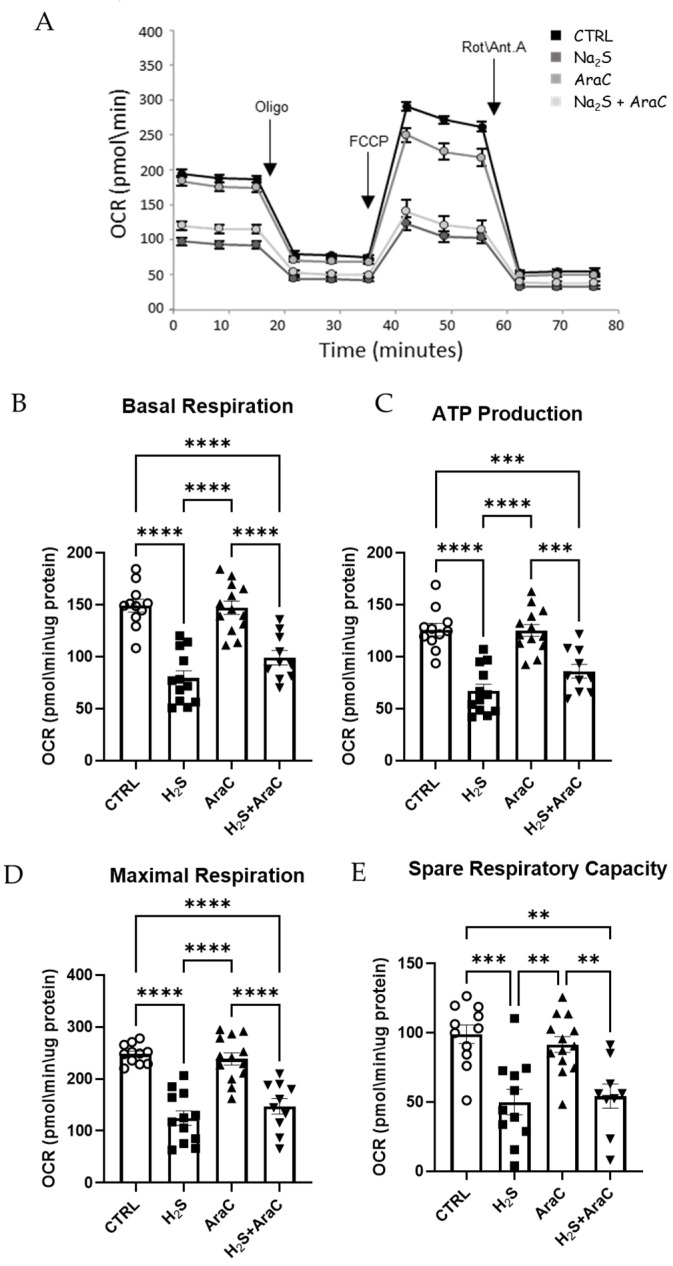
H_2_S treatment affects mitochondrial functionality. Oxygen consumption rate (OCR) was assessed through a Cell Mito Stress Test. (**A**) Representative mitochondrial stress test profile obtained with sequential injection (as indicated by the arrows) of oligomycin, FCCP, rotenone, and antimycin A. The x-axis represents the time points when each measurement was captured. The histograms represent the individual parameters: (**B**) basal respiration (**C**) ATP production, (**D**) maximal respiration, and (**E**) spare respiration capacity. All data were analyzed using the XFe Wave software 2.6. Data represent means ± SD of relative values vs. CTRL from seven independent experiments. ** *p* < 0.01; *** *p* < 0.001; **** *p* < 0.0001 statistical analysis was performed with one-way ANOVA with Tukey’s test for multiple comparisons. Circles, squares and triangles represent the different treatments, control, H_2_S, AraC, H_2_S + AraC.

A seahorse bioanalyzer was used to evaluate the mitochondrial metabolic activity (Figure 4A). To evaluate the toxicity of H_2_S on mitochondrial homeostasis, we analyzed mixed spinal cord cultures, with and without the addiction of Ara-C (to halt astrocyte proliferation), treated for 18 h with H_2_S (200 μM). We know from our previous works that this is a toxic protocol, especially for motor neurons [13]. We observed that every parameter measured by the assay, i.e., basal respiration (Figure 4B), ATP production (Figure 4C), maximal respiration (Figure 4D), and spare respiratory capacity (Figure 4E), was significantly altered by H_2_S. This latter evidence (observed after the addition of the proton ionophore FCCP) indicates the loss of ability of the cells to promptly respond to an increased energy demand.

**Figure 5:**
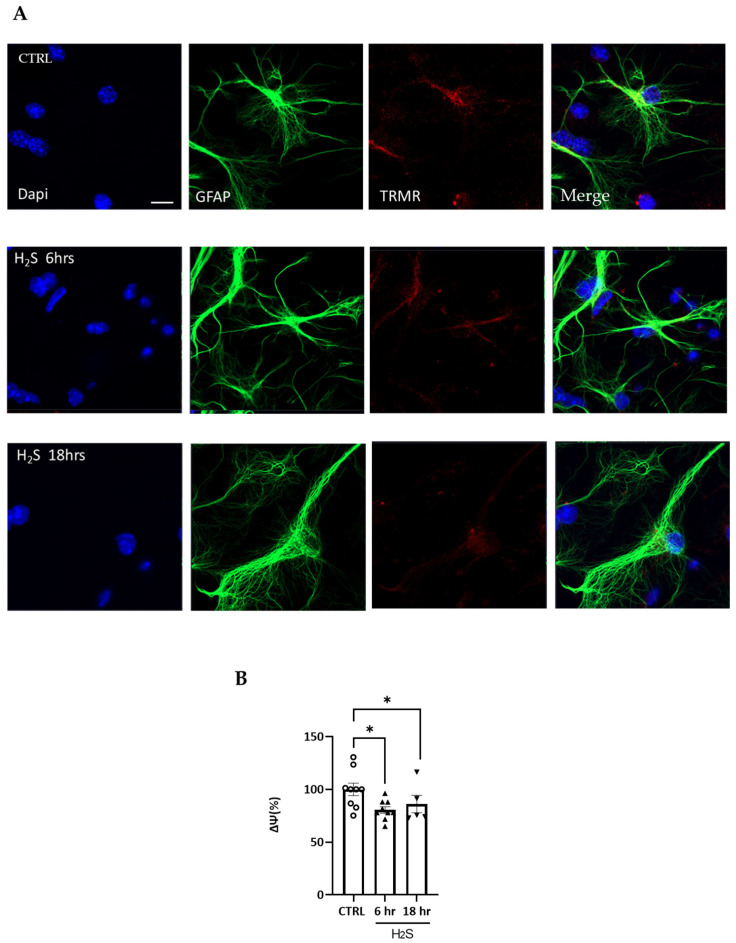
H_2_S alters mitochondrial membrane potential. H_2_S at 200 μM was incubated at different times, and then the cells were loaded with TRMR (red). The cells were fixed and incubated with Dapi and GFAP (astrocyte marker in green), and confocal images were captured at 40X. In A, non-treated cells are shown compared to cells treated for 6 and 18 h. The pixels values were obtained using Image J (Fiji software 2.9.0) (NIH, Bethesda, MD, USA) in the entire field. After 6 and 18 h of H_2_S incubation, there was also a decreased level of TRMR (red) in the astrocytes (**A**), which was also quantified (**B**). The mean of the pixels was quantified in at least three different fields in at least four slides for the cultures (*n* = 3). Data are presented as percentages normalized to non-treated values as mean ± SEM. All values were compared by a one-way ANOVA test with * *p* < 0.05. Scale bar: 10 micron (**A**).

Because of the comprehensive alteration in mitochondrial function, we sought to monitor the changes in mitochondrial membrane potentials (Δ*Ψ*m). To estimate the mitochondrial Δ*Ψ*m, we used the Δ*Ψ*m-dependent ratiometric indicator JC1. On the matrix side, the inner mitochondrial membrane retains a negative potential. Lipophilic molecules that are positively charged are trapped in the mitochondrial matrix and accumulate due to the membrane potential. The JC-1 monomers accumulate in the matrix and form aggregates, accompanied by a shifting in the absorption, resulting in a red emission peak. A reduction in the membrane potential results in a fluorescence change from red to green and a diffusion of the dye from the mitochondria to the cytosol [25]. Factors leading to the loss of the electrochemical gradient depolarize the membrane, triggering a loss of red fluorescence. In the control cultures, the vast majority of the mitochondria showed a high 600/530 ratio, indicative of an intact Δ*Ψ*m at all the time points considered (Supplementary Figure S3A,B). In the H_2_S-treated cultures, we observed a generalized and transient time-dependent Δ*Ψ*m reduction, showing a significant shift toward a lower 600/530 ratio at 6 h (Supplementary Figure S3B) and a recovery at 18 h. The 6 h shift was indicative of a reduction in the mitochondrial membrane electrochemical status in a significant subset of mitochondria during the time course. When we used an additional indicator of the membrane potential, TMRM, we measured a drop in Δ*Ψ*m at 6 and 18 h (Figure 5). It is noteworthy that the TMRM signal was reduced at 6 and 18 h, as well as in the GFAP+ cells (as shown in Figure 5A,B), which further indicates a diminished Δ*Ψ*m in a significant number of mitochondria under H_2_S-mediated toxicity.

**Figure 6:**
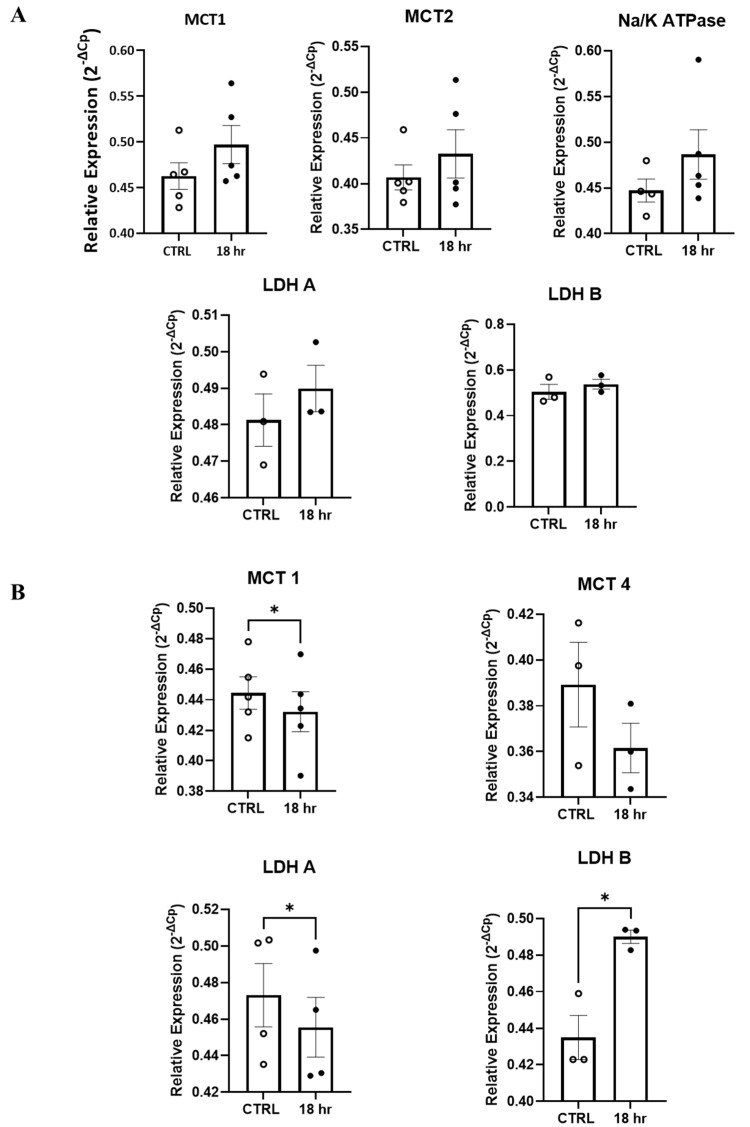
H_2_S promotes changes in the mRNA expression of components of lactate transport and the lactate/pyruvate metabolism. Primary spinal cord cultures were incubated with H_2_S (200 µM) for 18 h. (**A**) The mRNA expression of MCT1, MCT2, MCT4, LDHA, LDHB, and Na^+^/K^+^ ATP-ase α2 subunit was assessed in the whole culture. The tested mRNA expression remained unchanged, and we were not able to obtain a reliable quantification of MCT4. (**B**) Following the sorting of the ACSA1/2+ cells, the groups were compared using Student’s *t* test with * *p* < 0.05. White and black circles represent control and H_2_S (200 µM) 18 h.

Furthermore, given the effects of H_2_S on mitochondrial metabolism and mitochondrial membrane potentials, affecting, in turn, cellular metabolism [32,33] and the central role that glial cells play in the brain cellular metabolism [34,35], we decided to investigate if the expression levels of the components of the “astrocytes-to-neuron” lactate shuttle [36] were modified by the H_2_S treatment. The mRNAs encoding for the monocarboxylate transporters 1, 2, and 4 (*MCT1*, *MCT2*, and *MCT4*), for lactate dehydrogenase A and B (LDH-A and LDH-B), and for the *Na^+^/K^+^ ATP-ase α2* subunit were analyzed in the whole culture and in the sorted ACSA1 and 2+ cells. The qRt-PCR (Figure 6A) of the whole culture did not demonstrate any significant difference in the analyzed mRNAs between the control (CTRL) and H_2_S (18) samples. However, when we performed a similar analysis on the ACSA1+-/2+-sorted cells, we measured a significant decrease in the mRNAs encoding for MCT1 and LDH-A and a significant increase in the mRNA encoding for LDH-B (Figure 6B), while MCT2 mRNA did not change. Taken together, with the additional parameters related to mitochondrial function and metabolism, these observations suggest that our toxic protocol strongly affected mitochondrial homeostasis as well as monocarboxylate transport and production.

We performed a similar analysis and quantified the mRNAs by qRT-PCR, and the astrocyte-enriched samples revealed a significant decrease in MTC1 and LDHA, a significant increase in LDHB, and a decreasing trend of MCT4 mRNA. All values are presented as means ± SEM and were compared by using a one-way ANOVA test with * *p* < 0.05.

### 3.4. Hydrogen Sulfide Activates Death by Apoptosis

**Figure 7:**
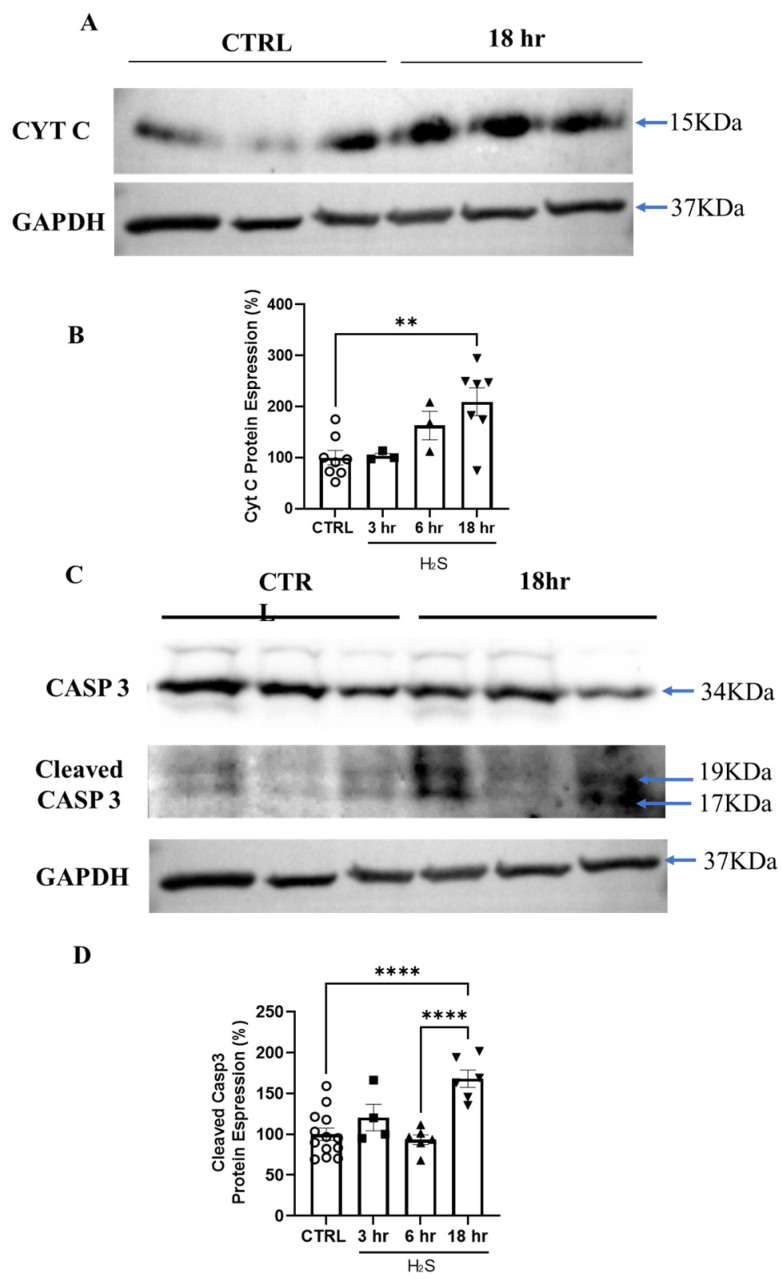
H_2_S stimulates CytC release and caspase-3 cleavage. Primary spinal cord cultures were incubated with H_2_S (200 µM) at the indicated times. (**A**) CytC protein bands in total cell extracts and glyceraldehyde 3-phosphate dehydrogenase (GAPDH) were used as a loading control. (**B**) The histogram shows the quantification of the H_2_S-induced CytC release as a percentage. The values (normalized to GAPDH) are expressed as mean ± SEM from three experiments. All values were compared by a one-way ANOVA test with ** *p* < 0.01. (**C**) Caspase-3 (CASP3) protein bands (34 KDa) are shown in the upper panel, and cleaved CASP3 (19 KDa and 17 KDa) is shown in the lower panel. (**D**) The values (normalized to GAPDH) are expressed as mean ± SEM from three experiments. All values were compared by a one-way ANOVA test with **** *p* < 0.0001. White circles, black squares and triangles represent the different time of treatments, control, 3, 6, 18 h of H_2_S.

We have previously demonstrated that the H_2_S toxicity is supported, at least in part, by a BAX-mediated mechanism [18]. The release of cytochrome c from mitochondria is downstream of BAX [37,38,39], and caspase-3, acting downstream of BAX, plays a key role in the execution of apoptosis [40,41]. Hence, we decided to investigate whether the H_2_S treatment promoted apoptosis via the activation of the intrinsic pathway [42]. We analyzed the activation of BAX, leading to the release of cytochrome c and the cleavage of caspase-3. Primary cultures were incubated in the presence or absence of H_2_S for 3, 6, and 18 h. The time-dependent increase in BAX (Supplementary Figure S4) and the release of cytochrome c was determined by Western blotting. We measured a significant increase in BAX (Supplementary Figure S4A,B) and cytochrome c in the H_2_S-treated culture at 18 h (Figure 7A,B). The cytosolic increase (i.e., mitochondrial release) of cytochrome c was further corroborated by immunocytochemistry (Supplementary Figure S5A,B). Caspase-3, one of the major effectors of apoptosis, and its cleavage indicates the irreversible activation of cellular apoptosis. As shown in the figure, the caspase-3 cleavage reached significance after 18 h of H_2_S treatment (Figure 7C,D).

## 4. Discussion

Most of the biomolecules present in our body possess both physiological and pathological roles. Hydrogen sulfide is no exception, having a dual action either as being a neuroprotective and anti-inflammatory agent or a neurotoxic and pro-inflammatory agent [5]. Therefore, the purpose of this study was to gain additional insight into our understanding of its cellular actions, paying particular attention to its effects on astrocytes.

Moving from being merely considered a cellular layer filling between and gluing neurons, astrocytes have become recognized for their fundamental role in controlling CNS homeostasis, acting as neurotransmitter recyclers, providing nourishment for neurons, and operating to provide function and stability to the neuronal networks [43,44,45]. Indeed, astrocytes are extremely reactive cells that actively respond to CNS challenges, and they are now a hallmark of many neuro-pathologies, like ALS [46,47,48,49,50]. Reactive astrocytes can change their shape as well as their morphology, leading to different patterns of secretion from anti-inflammatory to pro-inflammatory and toxic factors [51]. As demonstrated by us and others [4,13], H_2_S is released by astrocytes. Here, we pinpoint its ability to affect glial cells connecting the network and promoting their transformation to distinct subtypes without changing their overall number. Our results indicate that H_2_S functions as an inflammatory mediator, as is suggested by the increase in the body volume and by the switch from the A2 to the A1 astrocytic subtype, despite not affecting GFAP expression and the astrocytic cell count. These observations are in line with a theory picturing H_2_S as one of the factors released by glial cells [4] in a toxic environment that, in turn, smooth the transition of astrocytes to a reactive form, which consecutively worsens the same toxic environment.

The effect of H_2_S on astrocytic activation was further confirmed by the Cx43 protein data. Almad et al. linked the increase in Cx43 hemichannels with astrocytic-mediated toxicity to motor neurons in ALS [52,53]. As previously demonstrated by us and confirmed here, H_2_S is toxic to motor neurons, and we reported that its levels are increased in the cerebrospinal fluid of ALS patients and in an animal model SOD1G93A [13,54]. Hence, similarly to NO [55], H_2_S also leads to phenotypic changes in astrocytes toward a reactive phenotype and the upregulation of Cx43. Cxs are principal constituents of gap junction channels and hemichannels, mediators of astrocyte communication with neighboring glial cells, and an extracellular medium that plays significant roles in physiological (tissue homeostasis and metabolic transport) and pathological (apoptosis) processes [29,52,53,56]. It is noteworthy that increased Cx43 levels have also been described in Alzheimer’s disease and Parkinson’s disease and have been linked to the dysregulation and activation of astrocytes [57,58,59,60].

We then sought to postulate a potential mechanism for the H_2_S-induced toxic effect on astrocytes by modulating mitochondrial bioenergetics. Here, we showed that in primary spinal cord cultures, H_2_S-mediated toxicity was caused by the mitochondrial release of cytochrome c and the activation of caspase-3, as well as a time-dependent generalized disequilibrium in the mitochondrial membrane potential, an impairment in mitochondrial respiration parameters, and a consequent drop in ATP production. The activation of apoptosis is consistent with our data here and with a previous report, where we showed that H_2_S toxicity is partly reverted by the inhibition of a Bax-dependent mechanism [18]. Bax activation promotes the mitochondrial apoptotic pathway, which results in the release of cytochrome c and caspase-3 cleavage (key biochemical hallmarks of apoptosis). Nagai et al. [61] provided strong evidence that a Bax-dependent cell death pathway mediates non-cell autonomous motor neuron death in the pathology of ALS. Hence, as we have already proposed [14,18], these results further imply the participation of H_2_S as an astrocytic release factor in the non-cell autonomous degeneration described in ALS.

Mitochondrial bioenergetic data were analyzed with and without Ara-C to evaluate whether limiting the number of glial cells could have an impact on the overall mitochondrial homeostasis under H_2_S. We did not observe any effect on mitochondrial function due to the presence or absence of glial cells. All the parameters evaluated, i.e., basal respiration, maximal respiration, spare respiratory capacity, and ATP production, were decreased under the H_2_S treatment. Hence, we confirm that H_2_S toxicity is associated with compromised mitochondrial function, with reduced oxygen consumption, and with a sharp decrease in ATP production.

Because mitochondrial dysfunction leads to an abnormal metabolism [62], we investigated the effect of H_2_S on components of the lactate shuttle, which is a lactate-driven fuel source between neurons and astrocytes [63] and is composed of three monocarboxylate transporters (MCT1, MCT2, and MCT4), two lactate dehydrogenase subunits (LDH-A and LDH-B), and the Na^+^/K^+^ ATPase α2 subunit known to associate with MCT1 [27,64]. LDH-A has been associated with the conversion of pyruvate to lactate, while LDH-B is linked to the conversion of lactate to pyruvate [61]. From our data, which showed a significant increase in LDH-B accompanied by a significant decrease in LDH-A, we can infer that the H_2_S treatment favored a shift toward an increased production of pyruvate or a likely decrease in the pyruvate-to-lactate conversion, an important pathway in ATP generation [63,64,65]. Furthermore, the concurrent significant decrease in MTC1 and the decrease (although not significant) in MCT4 indicate that the transfer of the lactate dehydrogenase product from astrocytes to neurons was further compromised. Lactate is the end product of the glycolytic metabolism and a central substrate of energy metabolism for neurons [27]. Hence, we can envision a context in which H_2_S toxicity alters mitochondrial function and hampers lactate production and transport, thus depriving neurons of their essential fuel.

Furthermore, due to the active role of astrocytes in ALS-related motor neuron death and since neuroinflammation induces the formation of reactive astrocytes, which are considered harmful and known to induce the death of neurons and oligodendrocytes [66], our data showing a role for H_2_S in inducing an A2-to-A1 shift support the hypothesis of this gaseous mediator as an astrocytic agent that, together with other toxins, likely drives neuronal degeneration by increasing neuroinflammation and hampering metabolic supply to neurons.

Although the underlying mechanism(s) leading to the overproduction of H_2_S in ALS is yet to be determined, our findings provide further insights into the role of H_2_S as a pro-inflammatory agent released by astrocytes and that acts as a paracrine/autocrine mediator in glial cells, affecting the reactivity of astrocytes and their ability to nurture motor neurons.

## Figures and Tables

**Table 1:** Antibodies used in this study.

Antibody	Source	(#CAT)	Dilution
Alfa-Tubulin	Sigma-Aldrich (Milan, Italy)	(T6199)	1:500 (IF)
Caspase-3	Invitrogen (Rodano, Italy)	(PA5-77887)	1:100 (WB)
Bax	Abcam (Cambridge, UK)	(Ab5714)	1:1000 (WB)
Connexin 43	Sigma-Aldrich	(C6219)	1:500 (WB)
			1:200 (IF)
Cytocrome C	Invitrogen	(33-8500)	1:100 (WB)
Galectin 3	Invitrogen	(A3A12)	1:200 (IF)
GAPDH	Calbiochem (Milan, Italy)	(CB1001)	1: 60.000 (WB)
GFAP	Immunological Sciences (Rome, Italy)	(AB-10678)	1:500 (IF)
IBA 1	Wako (DBA, Milan, Italy)	(019-19741)	1:500 (IF)
SMI 32	Covance (Campoverde, Milan, Italy)	(SM1-32P)	1:500 (IF)
S100B	Sigma-Aldrich	(SAB5700647)	1:100 (IF)

**Table 2:** Primer sequences used for qRT-PCR.

Name	Forward Sequence	Reverse Sequence
LDHA NM-010699.2	CAGTGGCTTTGCCAAAAACCGAGT	CCATCAGGTAACGGAACCGCG
LDHB NM-008492.3	CCTGCTGACTTTGCAGTGGCTCC	TCGCCGCGGCAGCCTCATCAT
MCT1 NM-009196.4	TTGGACCCCAGAGGTTCTCC	AGGCGGCCTAAAAGTGGTG
MCT2 NM-001428808.1	CAGCAACAGCGTGATAGAGCTT	TGGTTGCAGGTTGAATGCTAAT
MCT4 NM-001038653.1	CGGCTGGCGGTAACAGAGTA	CGGCCTCGGACCTGAGTATT
Na^+^/K^+^-ATPase α2 NM-178405.3	GAGACGCGCAATATCTGTTTCTT	ACCTGTGGCAATCACAATGC
CX 43 NM-010288.4	CCCGAACTCTCCTTTTCCTT	TGGGCACCTCTCTTTCACTT
Ribosomal protein L34 NM-001005859.4	GGTGCTCAGAGGCACTCAGGATG	GTGCTTTCCCAACCTTCTGGTG

## Data Availability

Article distributed under the terms and conditions of the Creative Commons Attribution License (http://creativecommons.org/licenses/by/4.0/, accessed on 10 October 2024).

## Supplementary Materials

The following supporting information can be downloaded at: https://www.mdpi.com/article/10.3390/antiox13101241/s1, Figure S1: (A) Percentage of ACSA-2^+^ cells (astrocytes) and CD90.2+ cells (neurons) under H_2_S treatment. While ACSA-2^+^ were not affected by H2S toxicity, the number of CD90.2+ cells decreased significantly at 18 h. (B) H_2_S toxicity toward motor neurons was confirmed by direct counting of the SMI32+ neurons, an equimolar treatment with NaCl did not show any significant toxicity; Figure S2: (A) Representative images of fibroblast-like (1), arborized (2) and polarized (3) astrocytes morphologies after GFAP (green) staining. (B) The different shapes were quantified by direct counting of the GFAP+ cells in Control (Ctrl) and H_2_S treated cultures at 3, 6 and 18 h. (**C**) Representative western blot of the GFAP expression levels at the indicated times; Figure S3: (A) Immunofluorescence of spinal cord culture in Control (upper panels) and under H_2_S toxicity (lower panels, 6 h and 18 h treatment). The cultures were challenged at different times with H_2_S 200 μM, and then loaded with the mitochondrial membrane potential dye JC-1, fixed and probed with DAPI. (B) Quantitative analyses of the ratio between the JC-1 aggregate (red) and the JC-1 monomer (green) is presented as a percentage normalized to the non-treated cultures; Figure S4: H_2_S treatment provoked a time-dependent increase of BAX reaching significance at three hours of 200 μM H_2_S incubation. (A) Representative western blot showing the Bax increase after three hours of H2S treatment, and a subsequent decrease after 18 h. (B) Data are the result of three independent experiments mean  ±  SEM; Figure S5: H_2_S treatment provoked the release of cytochrome c after 18 h of incubation. H_2_S 200 μM was incubated for 18 h, the cells were then fixed and probed with cytochrome c (green) and Dapi. Confocal images were capture with a 63× objective from three different cultures. Six images/cultures for each group were taken and the pixels were quantified using ImageJ (C:\Fiji.app\ImageJ-win64.exe) (NIH, Bethesda, MD, USA). (A) Representative images of Control (Ctrl) and treated cultures (18 h). (B) The graph indicates the cytosolic levels (in pixels) of cytochrome c reaching significance at 18 h.
