# Supplementary material for: Hydrogen Sulfide Modulates Astrocytic Toxicity in Mouse Spinal Cord Cultures: Implications for Amyotrophic Lateral Sclerosis
Source: Antioxidants (Basel). 2024 Oct 15;13(10):1241. doi: 10.3390/antiox13101241 (PMC11504967; doi:10.3390/antiox13101241)
Supplement: Supplementary file 1 [file antioxidants-13-01241-s001.zip › antioxidants-3181372-supplementary.pdf]

A

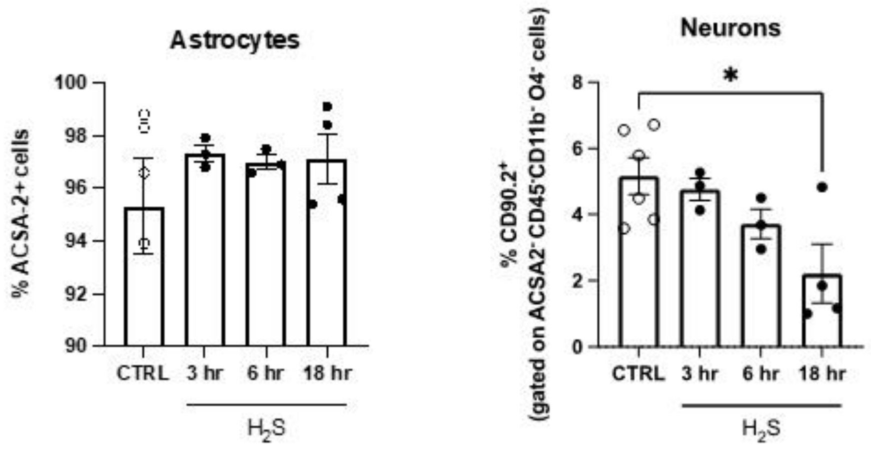

B

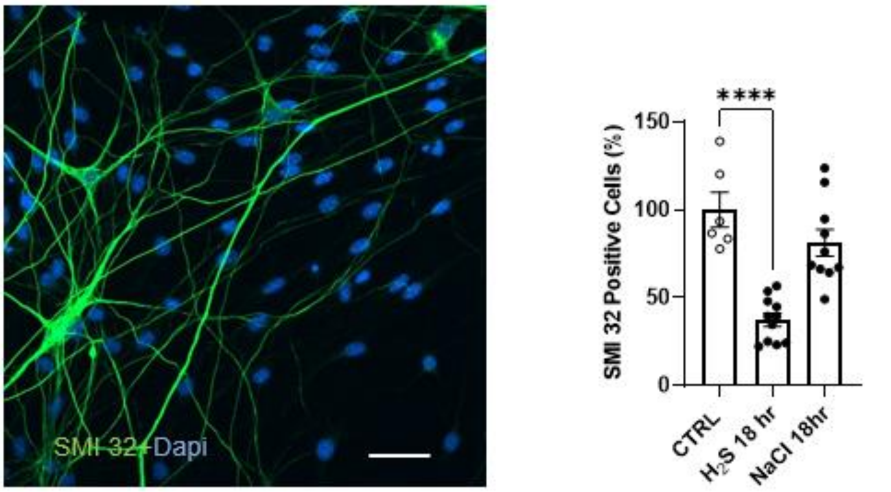

Supplementary Figure S1. (**A**) Percentage of ACSA-2+ cells (astrocytes) and CD90.2+ cells (neurons) under H_2S treatment. While ACSA-2+ were not affected by H2S toxicity, the number of CD90.2+ cells decreased significantly at 18 h. (**B**) H_2S toxicity toward motor neurons was confirmed by direct counting of the SMI32+ neurons, an equimolar treatment with NaCl did not show any significant toxiciy. All values were compared by using one-way ANOVA with * $p < 0.05$, **** $p < 0.0001$ (Scale Bar 20 μm).

A

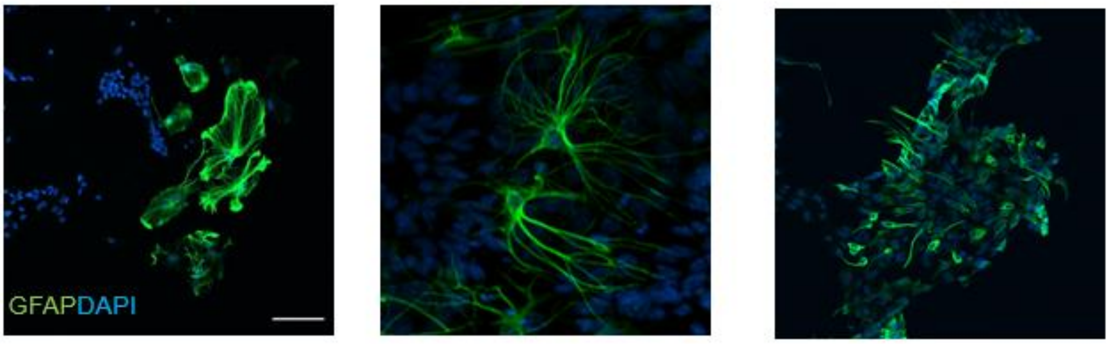

B

100
micrmetri

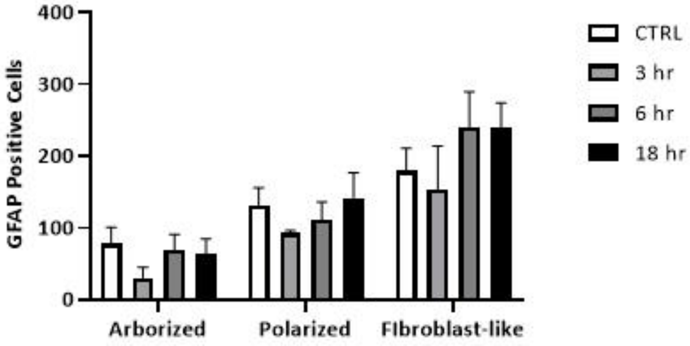

C

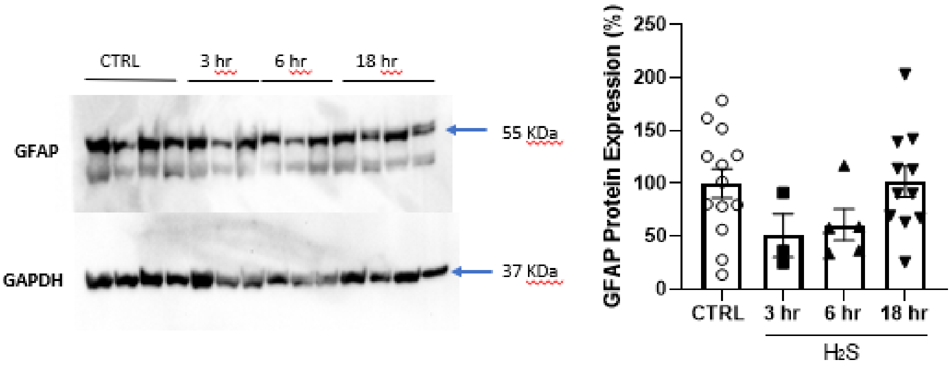

Supplementary Figure S2. (**A**) Representative images of fibroblast-like (1), arborized (2) and polarized (3) astrocytes morphologies after GFAP (green) staining. (**B**) The different shapes were quantified by direct counting of the GFAP+ cells in Control (Ctrl) and H_2S treated cultures at 3, 6 and 18 h. (**C**) Representative western blot of the GFAP expression levels at the indicated times. Data are expressed as mean ±SEM (Scale Bar 20 μm).

A

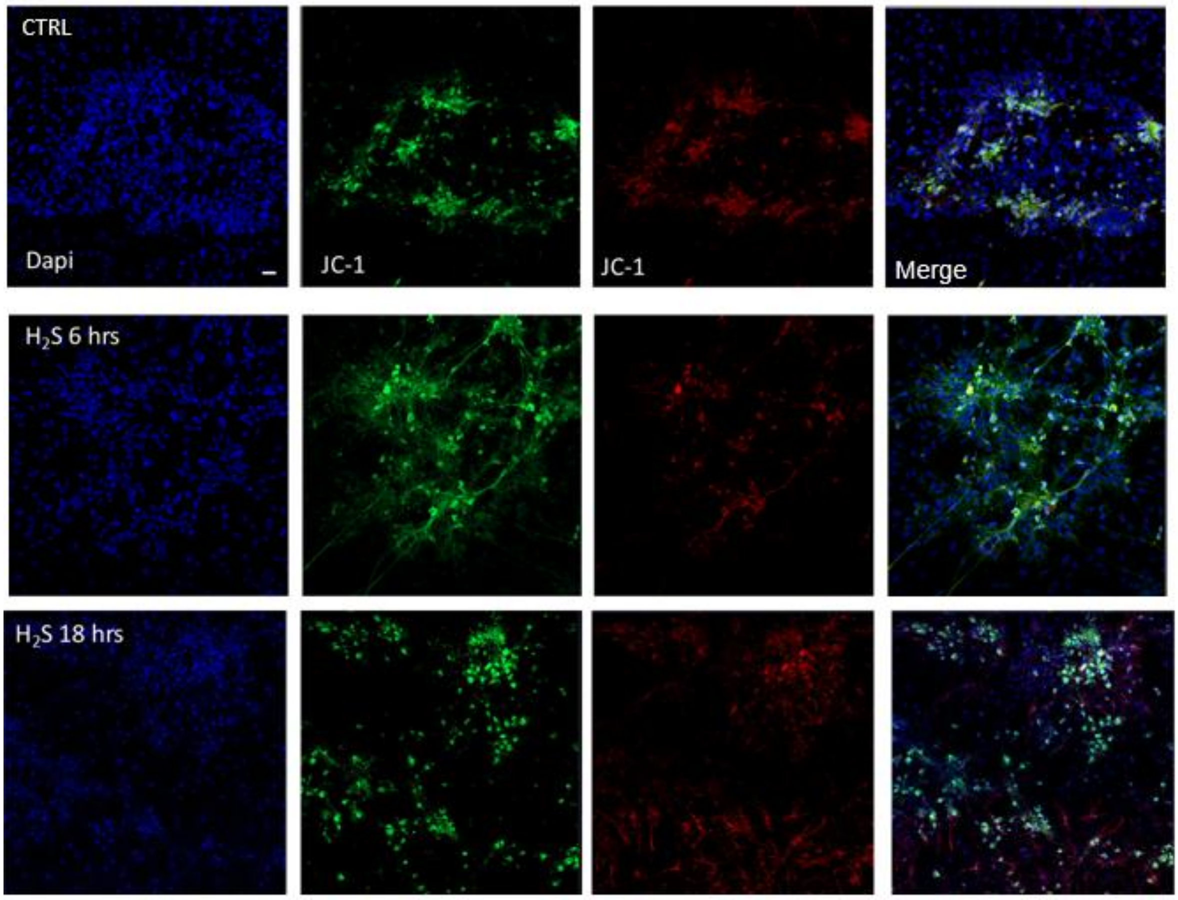

B

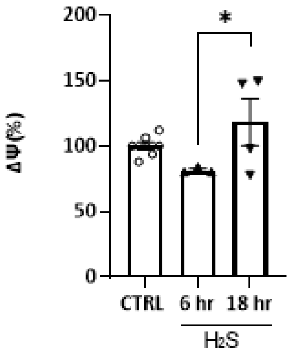

Supplementary Figure S3. (**A**) Immunofluorescence of spinal cord culture in Control (upper panels) and under H_2S toxicity (lower panels, 6 h and 18 h treatment). The cultures were challenged at different times with H_2S 200 μM, and then loaded with the mitochondrial membrane potential dye JC-1, fixed and probed with DAPI. (**B**) Quantitative analyses of the ratio between the JC-1 aggregate (red) and the JC-1 monomer (green) is presented as a percentage normalized to the non-treated cultures. Data are expressed as mean ±SEM of three fields from three separate experiments. The values were compared by using one-way ANOVA with * $p < 0.05$ (Scale bar 20 μm).

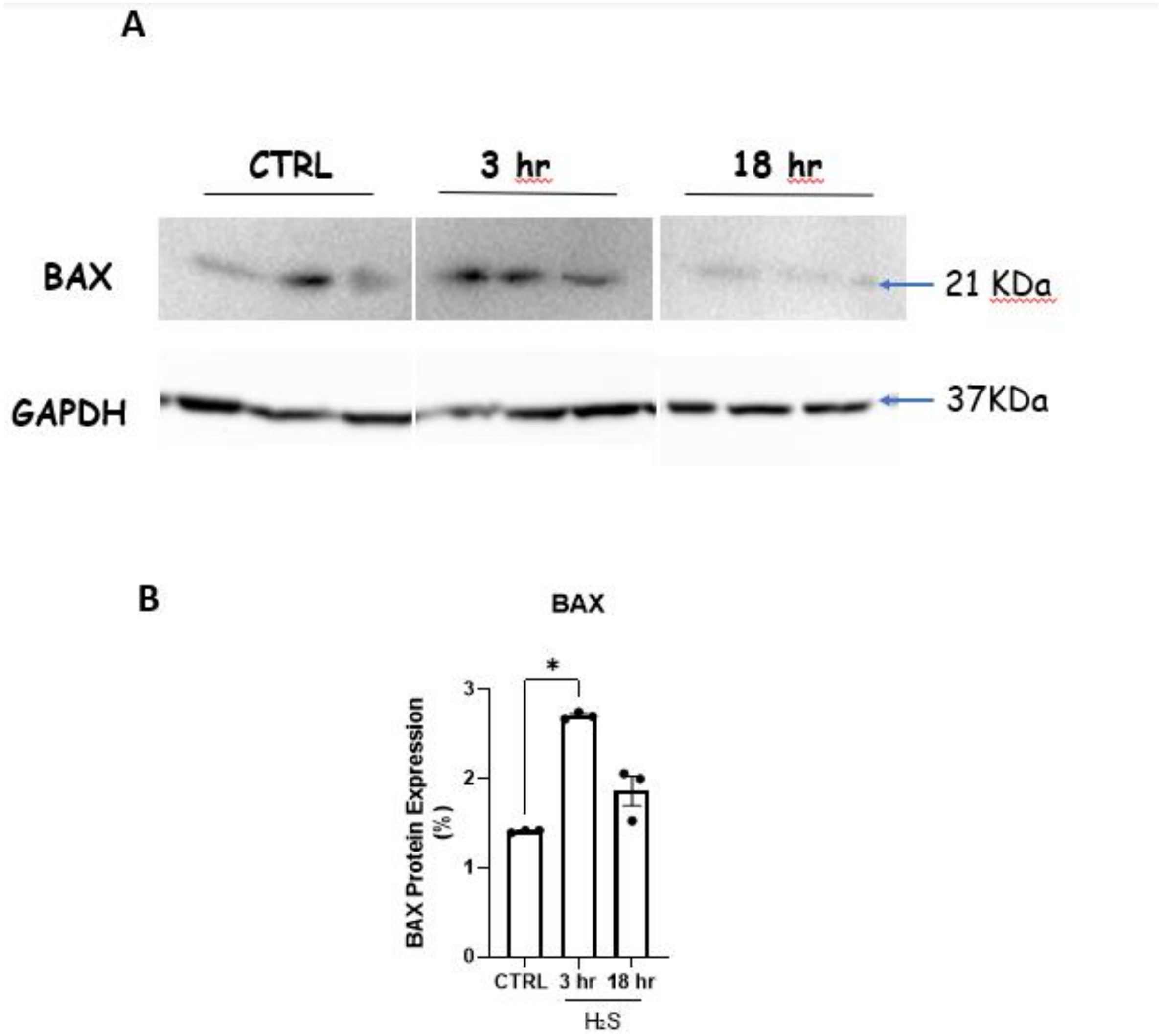

Supplementary Figure S4. H_2S treatment provoked a time-dependent increase of BAX reaching significance at three hours of 200 μM H_2S incubation. (**A**) Representative western blot showing the Bax increase after three hours of H2S treatment, and a subsequent decrease after 18 h. (**B**) Data are the result of three independent experiments mean ± SEM. All values were compared by using one-way ANOVA with * $p < 0.05$.

A

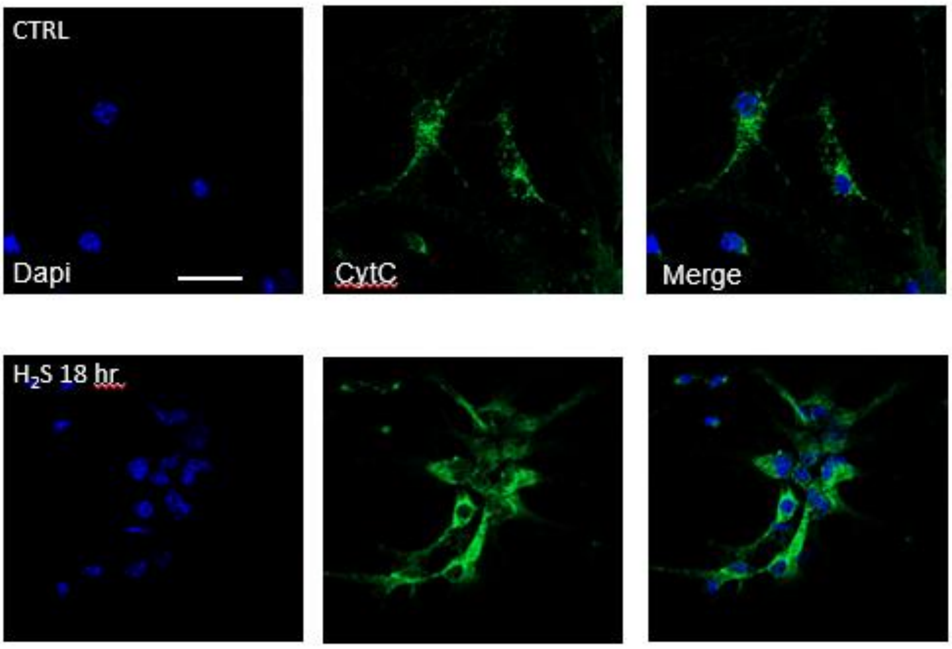

B

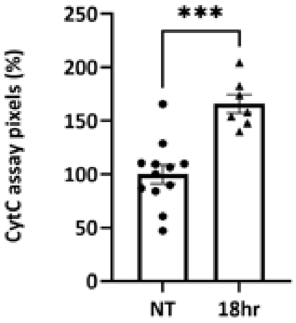

Supplementary Figure S5. H_2S treatment provoked the release of cytochrome c after 18 h of incubation. H_2S 200 μM was incubated for 18 h, the cells were then fixed and probed with cytochrome c (green) and Dapi. Confocal images were capture with a 63X objective from three different cultures. Six images/cultures for each group were taken and the pixels were quantified using Image J (Fiji software). (**A**) Representative images of Control (Ctrl) and treated cultures (18 h). (**B**) The graph indicates the cytosolic levels (in pixels) of cytochrome c reaching significance at 18 h. The values of the pixels are presented as percentage normalized to the non-treated values as mean ± SEM (**B**). All values were compared by using one-way ANOVA with *** $p < 0.001$ (Scale Bar 20 μm).
